# Beauty versus the beast: The UK public prefers less‐extreme body shapes in brachycephalic dog breeds

**DOI:** 10.1002/vetr.5671

**Published:** 2025-07-04

**Authors:** Elizabeth Youens, Dan G. O'Neill, Zoe Belshaw, Sayaka Mochizuki, Johanna Neufuss, Mickey S. Tivers, Rowena M. A. Packer

**Affiliations:** ^1^ Clinical Science and Services Royal Veterinary College Hatfield UK; ^2^ Pathobiology and Population Sciences Royal Veterinary College Hatfield UK; ^3^ EviVet Evidence‐Based Veterinary Consultancy Nottingham UK; ^4^ Blue Cross Burford UK; ^5^ Paragon Veterinary Referrals Wakefield UK

## Abstract

**Background:**

Brachycephalic dog breeds often exhibit a suite of extreme conformations linked with several severe and long‐term disorders. Despite this, ownership levels of brachycephalic breeds remain high internationally, with physical appearance being an important driver in their acquisition. Moving these breeds away from conformational extremes is necessary to protect canine welfare. This study aimed to explore how a range of extreme conformations in common brachycephalic breeds are perceived by the UK public.

**Methods:**

An online questionnaire used images generated by artificial intelligence of typical, less extreme and super extreme versions of three common brachycephalic breeds (French Bulldog, Pug and English Bulldog) to assess preferences among the UK general public for varying degrees of conformational extremes of muzzle length, eye size/shape, skin wrinkling and tail length.

**Results:**

The survey included results from 4899 participants. The less extreme versions of all three breeds were rated significantly higher by the public, including owners of purebred brachycephalic dogs, for attractiveness, perceived health, how happy they made the participant feel, ethics of breeding and how much the participant would like to own that dog (*p* < 0.001).

**Limitations:**

The participants were UK‐based only and largely female, restricting generalisability.

**Conclusion:**

If given a choice, the UK public prefers less extreme body shapes in brachycephalic dog breeds. This presents major welfare opportunities to reverse the current normalisation of extreme body shapes in some currently popular brachycephalic dog breeds.

## INTRODUCTION

Ownership of brachycephalic (short‐muzzled) breeds has risen and remained high over the last two decades, with their distinctive, extreme facial and body conformation regularly promoted in popular culture, such as advertising and films.^1^, ^2^ For example, new registrations of French Bulldogs with international kennel clubs have even surpassed those of persistently popular breeds, such as the Labrador Retriever, at some points in time. UK Kennel Club new registrations for French and English Bulldogs revealed a peak in registrations around 2021.^3^, ^4^, ^5^

However, despite many people perceiving the brachycephalic appearance as ‘cute’,^6^, ^7^, ^8^, ^9^ the brachycephalic skull shape is considered an extreme conformation. An extreme conformation is defined as a physical appearance that has been so significantly altered away from the ancestral natural canine appearance that affected dogs commonly suffer from poor health and welfare.^10^ There is now a large evidence base linking brachycephaly with several severe and chronic disorders, including brachycephalic obstructive airway syndrome (BOAS).^11^, ^12^, ^13^ BOAS affects the upper airway by obstructing airflow and leading to respiratory compromise, with the risk of BOAS increasing with shortening muzzle length.^11^, ^12^ This negatively affects canine welfare by placing limitations on natural day‐to‐day canine activities that are essential to comfort and that facilitate positive affective experiences such as sleep,^14^ exercise^15^ and heat tolerance.^16^ It also leads to increased negative affective experiences such as breathlessness.^17^ Other disorders associated with brachycephaly include ocular disease such as corneal ulceration,^18^, ^19^ keratoconjunctivitis sicca^20^ and prolapsed nictitating membrane gland,^21^ which can result in ocular pain, discomfort and reduced vision. Brachycephaly is also associated with skin conditions such as skin fold dermatitis,^22^ dystocia and subsequent high levels of caesarean sections,^23^ spinal disease^24^, ^25^ (in some cases as a result of DVL2 mutation^26^) and heat‐related illness.^27^ Many commonly owned brachycephalic breeds, such as the French Bulldog, Pug and English Bulldog, exhibit substantially increased odds of several of these conformation‐related diseases.^28^, ^29^, ^30^ Concerningly, despite the serious negative welfare impacts of these conditions on the dogs themselves, normalisation of clinical signs related to BOAS and several of these other conditions appears to be pervasive.^31^, ^32^, ^33^, ^34^

Physical appearance is an important driver of acquisition in dogs^35^ and has been demonstrated to be more influential on breed choice for owners of certain extreme brachycephalic breeds (French Bulldog, Pug and English Bulldog) compared to owners of non‐brachycephalic breeds.^36^, ^37^ The power of appearance for acquisition desire may be driven by biological effects in people. Brachycephaly in dogs mimics certain infantile features known as the ‘Kindchenschema’ (baby schema or childlikeness), which evoke feelings of nurturing and care that may lead to an instinctive attraction to those breeds for some people.^8^, ^9^, ^34^, ^36^, ^38^

Cultural drivers, such as fashion^39^ and the influence of the show ring within pedigree dog communities, are also likely to play a role in breed acquisition choice. Pedigree dogs are rewarded in the show ring based on their adherence to certain physical characteristics defined in breed standards or to the personal preferences of each individual judge. Despite all UK Kennel Club breed standards having a disclaimer that ‘breeders and judges should at all times be careful to avoid obvious conditions or exaggerations which would be detrimental in any way to the health, welfare or soundness of this breed’,^40^ there has been widespread criticism of some breed standards for promoting extreme conformation.^41^ For example, the UK Kennel Club's breed standard for French Bulldogs expressly advises a ‘slightly undershot’ jaw (prognathism), for English Bulldogs a ‘muzzle short and broad’ (brachycephaly) and for Pugs a tail ‘tightly curled’ (a trait linked with spinal disease),^40^ all of which are features that meet the criteria for being extreme conformations. Regrettably, normalisation of extreme conformation in breed standards perpetuates these harmful traits and discourages ethical breeders from selecting away from such extreme types of dogs to the extent needed to make substantial health improvements.

Moderating the typical conformations of breeds with extreme brachycephaly could reduce conformation‐related disease^11^, ^18^ and improve the welfare of affected dogs^3^ while still retaining the distinctive breed status^42^ and valued predictability^43^ ascribed to individual purebred dog breeds, which are traits considered desirable by some potential dog owners.^43^, ^44^, ^45^ Moving towards wider public acceptance of less extreme variants of currently extreme breeds (and rejection of more extreme conformations) is also critical to improve the current welfare crisis related to brachycephaly in dogs. However, little is known about owner preferences for varying degrees of brachycephaly in dogs, beyond the brachycephalic appearance itself generally being highly valued.^35^ In cats, less extreme facial morphology has previously been reported to be preferred by the public compared to the most extreme brachycephalic morphologies seen in this species, for example, extreme Persians.^46^ Conversely, a public preference towards brachycephaly has been reported in rabbits.^47^

Decisions surrounding dog acquisition are complex and nuanced, with factors such as the level of research conducted and sources consulted during pre‐purchase research and route of acquisition varying within the dog‐acquiring population.^48^ Factors demonstrated to be important in owners' choice of which breeder to purchase from included breeders who would allow viewing of the puppies’ dam and breeders who were felt to care about their dogs.^49^ Factors demonstrated to be sought after by owners when selecting a particular breed/crossbreed to purchase included good companionship, size suited to owner lifestyle, and their general health.^49^ However, physical appearance has been demonstrated to be of high importance in dog acquisition,^35^ particularly for owners of brachycephalic breeds, who rated appearance as more influential upon their breed choice than a breed being generally healthy or having a long life expectancy.^36^ Moreover, recent research indicates that owners of extreme brachycephalic dogs rate flat faces and short necks as more desirable physical features than owners of mildly to moderately brachycephalic dogs and non‐brachycephalic dogs, and owners of both extreme brachycephalic dogs and mildly to moderately brachycephalic dogs rate small size, wrinkled skin, short legs and short tails as more desirable than owners of non‐brachycephalic dogs.^33^

Understanding public preference for degrees of extreme conformations could help inform future work on shifting demand away from dogs with more extreme conformations. If prospective brachycephalic dog owners were shown to consider more moderate conformations equally or even more attractive than the current extreme versions for common breeds, then market forces could encourage breeders to respond responsibly and produce dogs with these healthier, less extreme body shapes, offering widened opportunities for both their popularity as a breeder and improved canine welfare.

This study aimed to determine how well less extreme variants of three common brachycephalic breeds (French Bulldog, Pug and English Bulldog) are received by the UK public. Specific objectives were to assess perceptions of attractiveness, health and ethical breeding across a range of body shapes in these three breeds and how these relate to participant characteristics (e.g., the type of dog they currently or previously owned). The study hypothesis was that the UK public prefers less extreme versions of French Bulldogs, Pugs and English Bulldogs over the current typical and super extreme versions.

## MATERIALS AND METHODS

An online survey was developed to explore motivations and perceptions around dog acquisition within the UK public. Ethical approval was granted from the Social Science Research Ethical Review Board at Royal Veterinary College (URN SR2023‐0162). Designed iteratively between the authors, the survey was hosted using SurveyMonkey software and publicly open from 18 January to 1 March 2024. The survey was open to all UK adults (≥18 years of age and UK residents), including past, present and prospective dog owners, and also people with no prior or planned dog ownership. Participants could exit the survey at any time during completion, but they could not withdraw after their response was submitted because the survey was anonymous.

The participants were recruited via multiple pathways. These included snowball sampling using advertising posters (Appendix 1) placed on various UK‐based dog‐focused groups on social media sites (Facebook and Instagram). In addition, UK veterinary practices engaged clients with the survey via social media and physical posters in waiting rooms, and a range of pet industry stakeholders, including animal welfare charities and large veterinary organisations, promoted the survey on their social media channels or direct email. The survey link was also emailed directly to 962 owners of Pugs and Pug‐cross dogs participating in a linked practical research study who had consented to being contacted regarding further research. The complete list of disseminators is shown in the Acknowledgements section.

**FIGURE 1:**
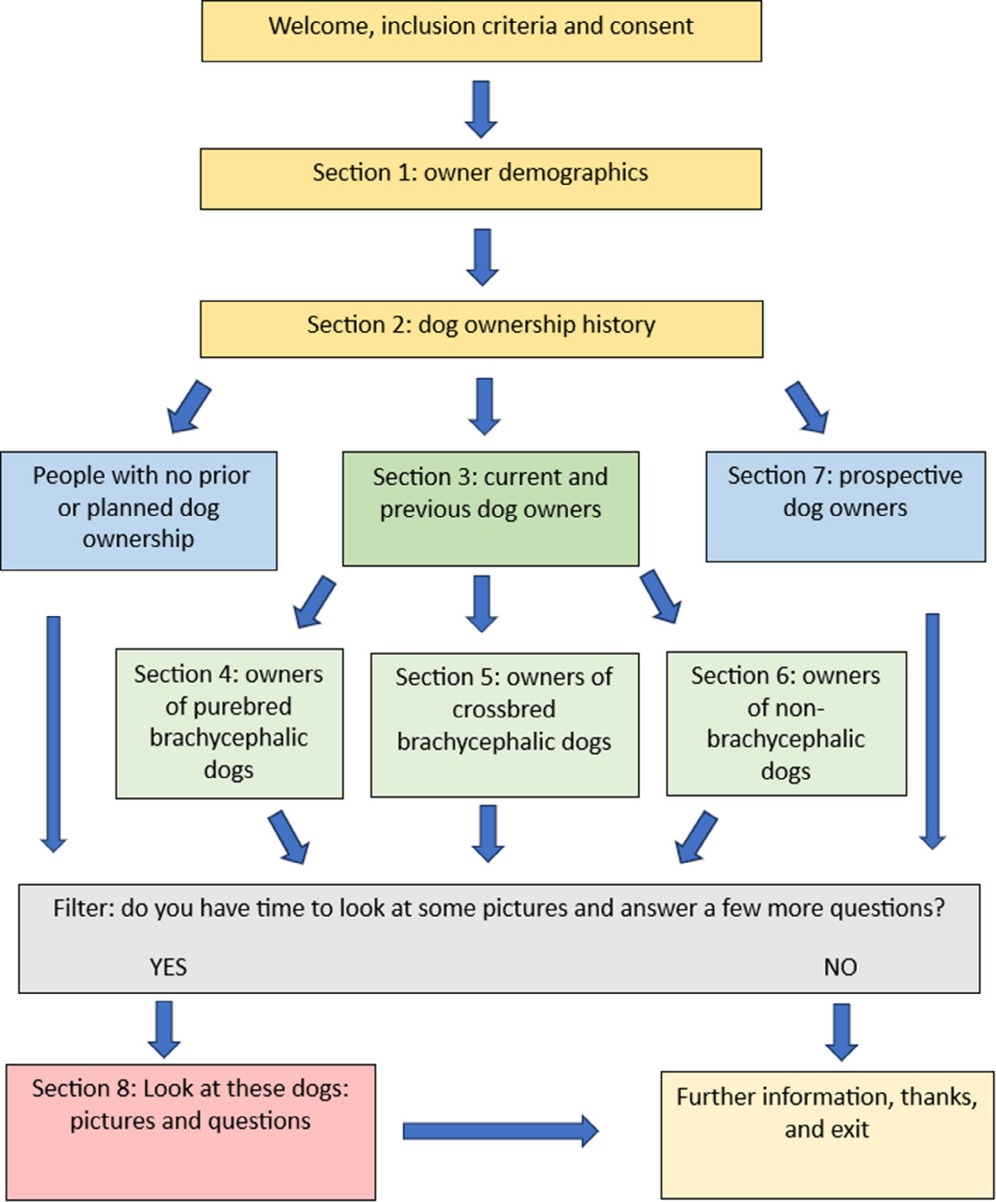
Schematic showing the structure of the survey exploring the UK public's perceptions of varying extremes of conformation in common brachycephalic breeds. All participants answered the first two sections and were then directed to further relevant sections depending on their answers using question logic.

The survey comprised of eight sections (Figure 1), with survey logic creating a bespoke experience for each participant based on their dog ownership history and type. The question types included closed‐choice questions (including binary yes/no, multiple choice and Likert‐scale questions) and open‐ended questions with free‐text boxes to facilitate qualitative analyses.

The first section captured owner demographics, including age, gender and household membership. The second section captured canine demographics, including the number and breed(s) of dog(s) currently and previously owned. When asked about their dog ownership, participants were provided with a definition of brachycephaly as ‘brachycephalic breeds are those which have shorter muzzles, and some also have flat/flattened faces’ and were provided with a list of common brachycephalic breeds. The term ‘purebred’ was defined as ‘both parents are of this same breed’ and ‘crossbred’ was defined as ‘a mix between two or more purebreds’. If, in section 3, the participants indicated that they currently owned, or had previously owned, a purebred brachycephalic dog, they were filtered to section 4. Those participants who currently or previously owned a crossbred brachycephalic dog were taken to section 5. All other dog owners (i.e., owners of non‐brachycephalic dogs, both purebred and crossbred) were taken to section 6. Participants who did not currently or had never previously owned a dog but were considering future acquisition were taken to section 7, with non‐dog owning participants who had no future intention to acquire a dog taken to section 8.

Sections 4‒7 explored pre‐acquisition motivations, achievement of expectations of dog ownership, attitudes towards crossbreeding and potential future acquisition choices, which are not reported here.

**FIGURE 2:**
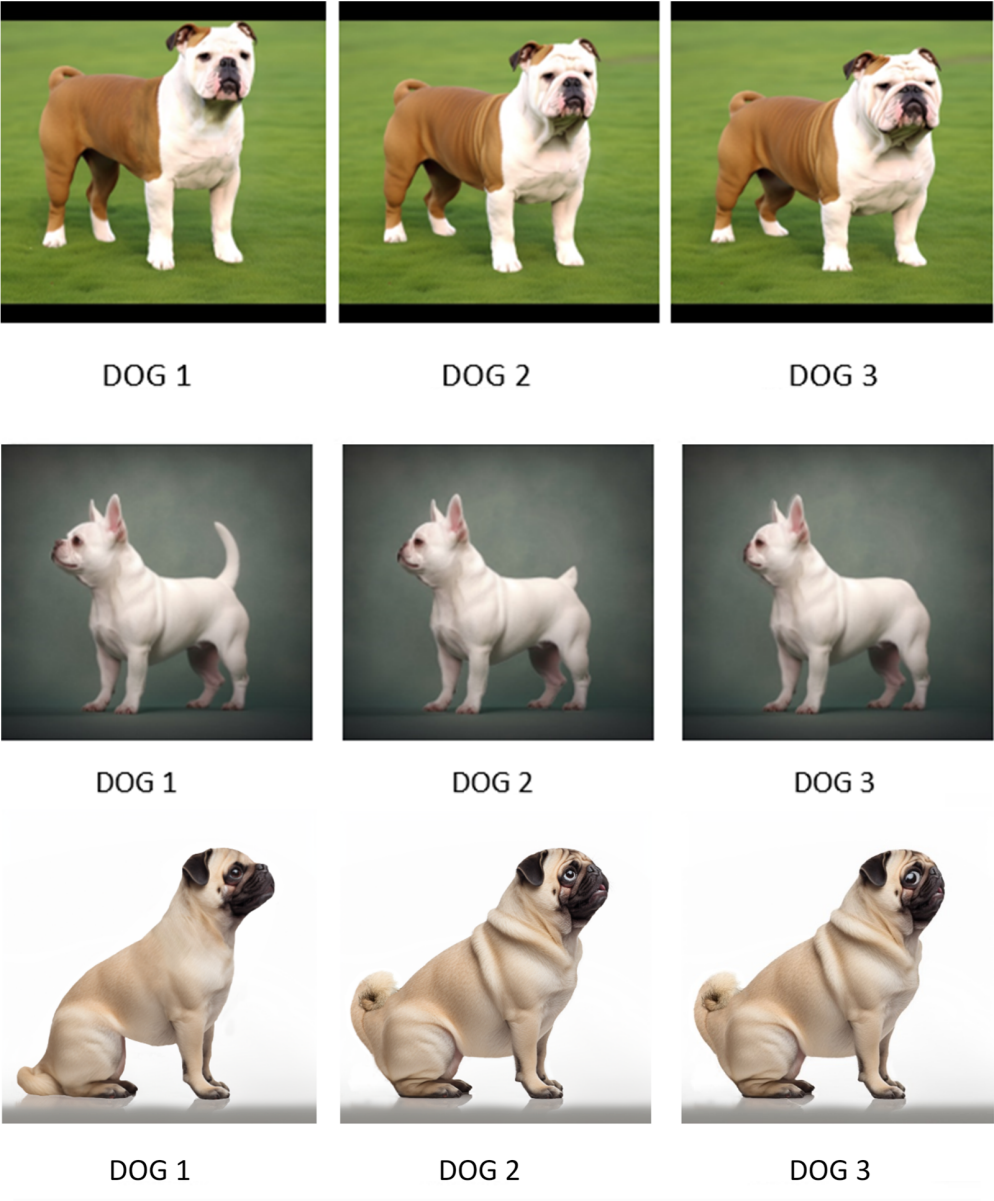
Sets of three images for each of the three most popular extreme brachycephalic breeds (French Bulldog, Pug and English Bulldog), which were generated and manipulated using a combination of artificial intelligence (AI) and digital editing to produce varying degrees of brachycephalic conformation and associated physical traits, for example, tail length/presence, degree of skin wrinkling. For each breed, dog 1 represents the less extreme conformation, dog 2 represents the current typical conformation and dog 3 represents the super extreme conformation.

The final section of the survey showed participants a series of three images of three commonly owned brachycephalic breeds: French Bulldog, Pug and English Bulldog. For each breed, these three images were generated and then manipulated by a combination of an artificial intelligence (AI) image generator (Midjourney; www.midjourney.com) and a digital image manipulation tool (Adobe Photoshop, version 23.2) to represent three variants of conformational extremeness: (1) typical for the breed currently, (2) less extreme (e.g., muzzle and tail manipulated to be longer and skin folds to be less pronounced), and (3) super extreme (e.g., muzzle and tail manipulated to be even shorter and skin folds even more pronounced) (Figure 2).

Visual access to images was given with every question to allow participants to repeatedly assess the images while deciding on their scores. The images were presented in the same order (less extreme, typical and then super extreme) for each question and each breed to reduce potential response bias created through inconsistent exposure^50^ and to aid efficiency of responses for participants, given the repeating nature of questions for each of the three breeds explored.

The participants were asked to score each of the three images showing varying degrees of extremeness for each breed from 1 (lowest) to 10 (highest) on five criteria:

1. How happy does each dog make you feel when you look at them?
2. How attractive does each dog look to you personally?
3. How healthy does each dog look to you personally?
4. How ethically do you think each of these dogs has been bred (i.e., in a way that prioritises health and temperament over appearance or financial gain)?
5. How much would you personally like to own each of these dogs?

Finally, participants were asked which of these dogs, if any, they thought were purebred French Bulldogs/Pugs/English Bulldogs. Participants had the option to choose ‘none of the above’ or any combination of dogs 1‒3 as being purebred.

Survey data were exported from SurveyMonkey into a Microsoft Excel (2023) spreadsheet and cleaned. Responses from ineligible participants were excluded (i.e., those who did not progress past the initial introductory information and/or consent, and duplicate responses). Data analysis was conducted in IBM SPSS Statistics (version 29.0.0.0).

Descriptive statistics (frequency and percentage) were reported. Chi‐squared testing was used to compare proportions between categorical variables. Likert data were analysed as continuous data,^51^, ^52^ and variation in mean scores between groups based on dog ownership type (purebred brachycephalic, crossbred brachycephalic and non‐brachycephalic) was compared using a one‐way ANOVA. Post hoc pairwise comparison with Fisher's least significant difference test was used when significant differences were detected between at least two groups. Image score data were compared at the univariable level using chi‐squared analysis for categorical variables and Mann‒Whitney *U*‐tests for non‐normally distributed continuous data (with data distribution ascertained by visual inspection of histograms). A multivariable generalised linear mixed model was built with image score as the outcome and participant number as a random effect to take into account this repeated effect. Variables liberally associated with image score in the univariable analyses (*p* < 0.2) were taken forward into the generalised linear model. Model development used backwards stepwise elimination, and the Hosmer–Lemeshow test statistic was used to evaluate model fit, with interactions between independent variables assessed for significance. Statistical significance was set at the 5% level.

## RESULTS

A total of 5035 responses were received, of which 4899 (97.3%) were eligible for inclusion in the final analysis. The participants predominantly self‐described as female (*n* = 4474, 91.3%), with 7.6% (*n* = 372) describing themselves as male, 0.5% (*n* = 24) describing themselves as non‐binary and 0.6% (*n* = 29) choosing ‘prefer not to say’. The most common age group of participants was 45‒54 years (*n* = 1095, 22.4%). Overall, 2865 participants (58.5%) lived with other adults but no children. Around one‐quarter of households included children or had children visiting regularly (*n* = 1179, 24.0%), and around one in six participants lived alone (*n* = 815, 16.6%). The most common type of home was a house with three or more bedrooms (*n* = 3415, 69.7%). There were 978 (20.0%) participants who worked in the animal sector, within which 354 (36.0%) worked as companion animal veterinarians or veterinary nurses. The full demographic data are reported in Appendix 2.

### Participant dog ownership

Around one‐quarter (*n* = 1270, 25.9%) of participants currently or previously owned a purebred brachycephalic dog, and 429 (8.8%) currently or previously owned a crossbred brachycephalic dog, while 3031 (61.9%) currently or previously owned a non‐brachycephalic dog. The most common number of dogs currently owned was one (*n* = 2524, 51.5%), with one‐quarter of participants (*n* = 1206, 24.6%) owning two. A minority of participants did not own a dog at the time of completing the survey but were considering owning one in the future (*n* = 130, 2.7%), or did not own a dog and had no intention of owning one in future (*n* = 39, 0.8%).

### AI image ratings

#### Levels of extreme conformation: public perceptions

In all three breeds studied (Pug, French Bulldog and English Bulldog), the score elicited on each of the five questions using rating scales differed significantly across the three degrees of extreme conformation (Figure 3). Across all question categories and including all participant responses, the less extreme dog consistently scored higher than the currently typical conformation dog, and the currently typical conformation dog scored higher than the super extreme conformation (Tables 1, 2, 3). The same effect was repeated across all three breeds (Tables 1, 2, 3).

FIGURE 3Clustered bar charts showing scores given by participants to each conformational variant of French Bulldogs, Pugs and English Bulldogs when asked: (a) How happy does each dog make you feel when you look at them? (b) How attractive does each dog look to you personally? (c) How healthy does each dog look to you personally? (d) How ethically do you think each of these dogs has been bred? (e) How much would you personally like to own each dog?

**Figure:**
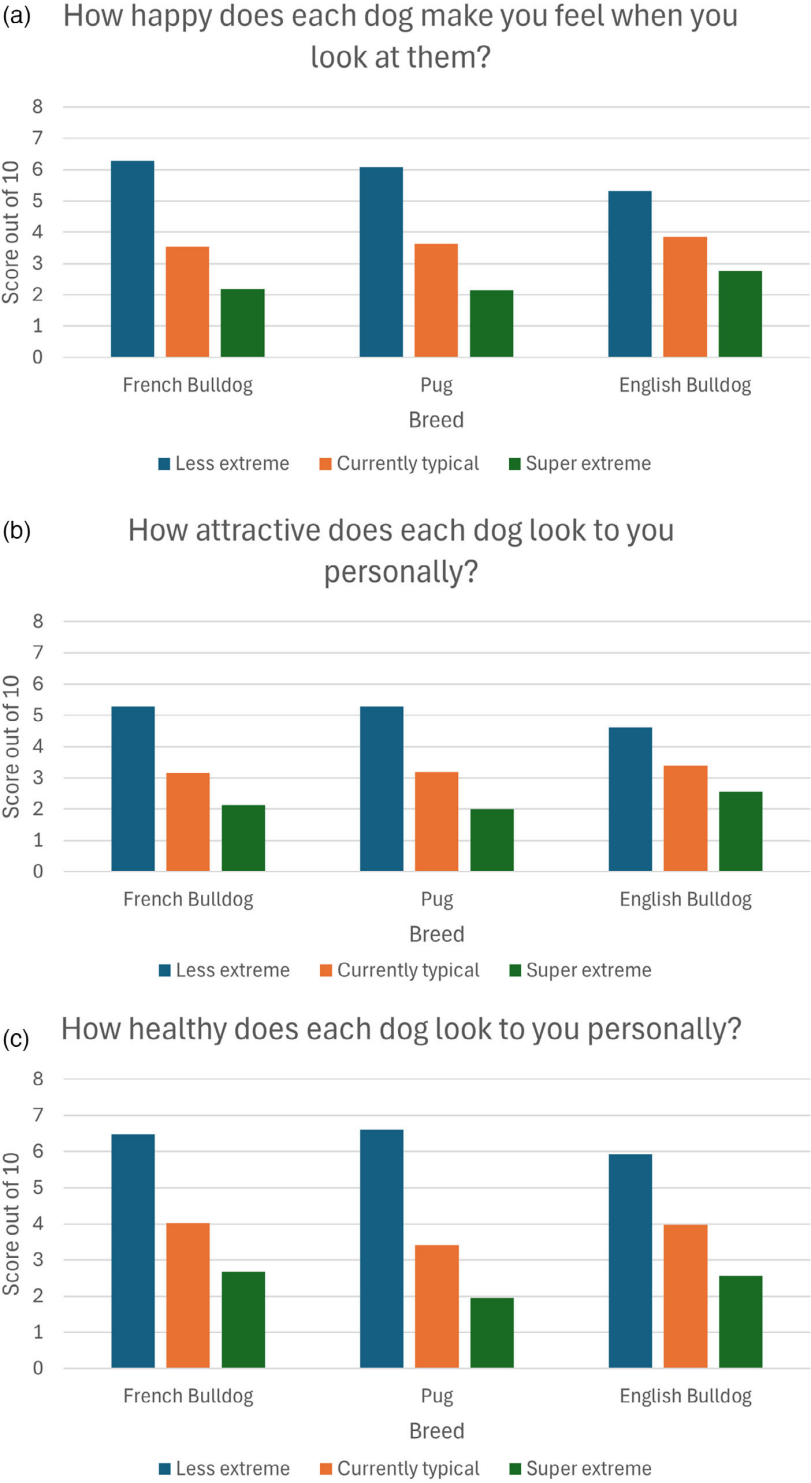

**Figure:**
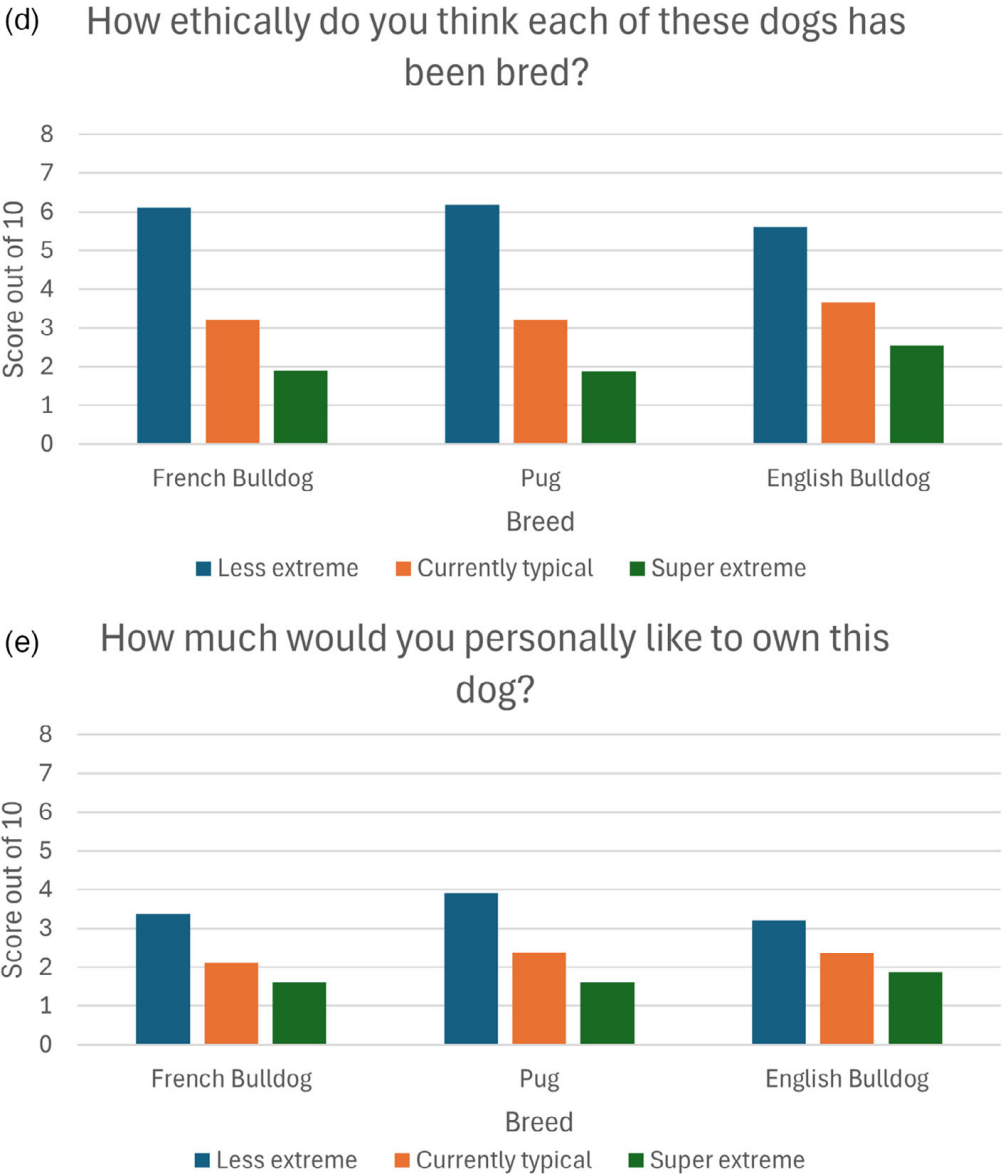

**TABLE 1:** Comparison of the rating scores given by the UK public to less extreme (LE), currently typical (CT) and super extreme (SE) conformations of French Bulldogs when asked a series of questions about the images.

	Mean score from 1 to 10 (standard deviation)					Post hoc comparison (LSD), *p*‐value		
French Bulldog	LE conformation	CT conformation	SE conformation	*F*‐statistic	One‐way ANOVA, *p*‐value	LE versus CT	LE versus SE	CT versus SE
How happy does each dog make you feel when you look at them?	6.29 (2.75)	3.55 (2.30)	2.17 (2.12)	2862.96	<0.001	<0.001	<0.001	<0.001
How attractive does each dog look to you personally?	5.28 (2.98)	3.16 (2.44)	2.14 (2.14)	1487.53	<0.001	<0.001	<0.001	<0.001
How healthy does each dog look to you personally?	6.48 (2.57)	4.03 (2.47)	2.68 (2.45)	2215.79	<0.001	<0.001	<0.001	<0.001
How ethically do you think each of these dogs has been bred?	6.10 (2.87)	3.20 (2.19)	1.89 (1.84)	3167.14	<0.001	<0.001	<0.001	<0.001
How much would you personally like to own each of these dogs?	3.38 (2.85)	2.11 (2.08)	1.61 (1.72)	606.15	<0.001	<0.001	<0.001	<0.001

Abbreviation: LSD, Fisher's least significant difference.

**TABLE 2:** Comparison of the rating scores given by the UK public to less extreme (LE), currently typical (CT) and super extreme (SE) conformations of Pugs when asked a series of questions about the images.

	Mean score from 1 to 10 (standard deviation)					Post hoc comparison (LSD), *p*‐value		
Pug	LE conformation	CT conformation	SE conformation	*F*‐statistic	One‐way ANOVA, *p*‐value	LE versus CT	LE versus SE	CT versus SE
How happy does each dog make you feel when you look at them	6.09 (2.70)	3.63 (2.63)	2.14 (2.14)	2315.86	<0.001	<0.001	<0.001	<0.001
How attractive does each dog look to you personally?	5.29 (2.89)	3.20 (2.68)	2.00 (2.04)	1533.41	<0.001	<0.001	<0.001	<0.001
How healthy does each dog look to you personally?	6.61 (2.54)	3.42 (2.36)	1.96 (1.87)	3972.05	<0.001	<0.001	<0.001	<0.001
How ethically do you think each of these dogs has been bred?	6.19 (2.78)	3.20 (2.39)	1.88 (1.87)	3129.80	<0.001	<0.001	<0.001	<0.001
How much would you personally like to own each of these dogs?	3.91 (2.97)	2.38 (2.51)	1.61 (1.78)	810.86	<0.001	<0.001	<0.001	<0.001

Abbreviation: LSD, Fisher's least significant difference.

**TABLE 3:** Comparison of the rating scores given by the UK public to less extreme (LE), currently typical (CT) and super extreme (SE) conformations of English Bulldogs when asked a series of questions about the images.

	Mean score from 1 to 10 (standard deviation)					Post hoc comparison (LSD), *p*‐value		
English Bulldog	LE conformation	CT conformation	SE conformation	*F*‐statistic	One‐way ANOVA, *p*‐value	LE versus CT	LE versus SE	CT versus SE
How happy does each dog make you feel when you look at them?	5.31 (2.77)	3.84 (2.58)	2.77 (2.58)	830.84	<0.001	<0.001	<0.001	<0.001
How attractive does each dog look to you personally?	4.61 (2.59)	3.39 (2.59)	2.56 (2.49)	534.07	<0.001	<0.001	<0.001	<0.001
How healthy does each dog look to you personally?	5.92 (2.59)	3.97 (2.40)	2.55 (2.24)	1736.92	<0.001	<0.001	<0.001	<0.001
How ethically do you think each of these dogs has been bred?	5.61 (2.77)	3.66 (2.39)	2.55 (2.36)	1355.97	<0.001	<0.001	<0.001	<0.001
How much would you personally like to own each of these dogs?	3.20 (2.75)	2.37 (2.27)	1.88 (2.02)	281.54	<0.001	<0.001	<0.001	<0.001

Abbreviation: LSD, Fisher's least significant difference.

#### Owners of different types of dogs: preferences for differing levels of extreme conformation

Across all three breeds, all questions elicited answers that were statistically significantly different between groups of owners (Tables 4, 5, 6). Owners of purebred brachycephalic dogs consistently scored all answers higher (i.e., more positively) across all degrees of conformational extremes compared to other owners.

**TABLE 4:** Comparison of the views of UK owners of purebred brachycephalic, crossbred brachycephalic and non‐brachycephalic dogs when asked a series of questions relating to images of French Bulldogs with varying levels of extreme conformation.

		Mean score from 1 to 10 (standard deviation)						Post hoc comparison (LSD), *p*‐value					
French Bulldog	Image category	Owners of purebred brachy‐cephalic dogs	Owners of crossbred brachy‐cephalic dogs	Owners of non‐brachy‐cephalic dogs	Non‐owners	*F*‐statistic	One‐way ANOVA, *p*‐value	Purebred versus crossbred brachy‐cephalic	Purebred versus non‐brachy‐cephalic	Purebred versus non‐owner	Crossbred versus non‐brachy‐cephalic	Crossbred versus non‐owner	Non‐brachy‐cephalic versus non‐owner
How happy does each dog make you feel when you look at them?	Less extreme conformation	7.62 (2.56)	7.35 (2.45)	5.69 (2.66)	6.34 (2.47)	132.36	**<0.001**	0.124	**<0.001**	**<0.001**	**<0.001**	**<0.001**	**0.002**
	Currently typical conformation	4.71 (2.68)	3.86 (2.21)	3.11 (2.04)	3.61 (1.92)	111.41	**<0.001**	**<0.001**	**<0.001**	**<0.001**	**<0.001**	0.242	**0.005**
	Super extreme conformation	2.94 (2.69)	2.27 (2.19)	1.89 (1.80)	2.26 (2.12)	53.60	**<0.001**	**<0.001**	**<0.001**	**<0.001**	**0.003**	0.957	**0.023**
How attractive does each dog look to you personally?	Less extreme conformation	6.84 (2.94)	6.42 (2.89)	4.59 (2.77)	5.42 (2.72)	152.65	**<0.001**	**0.024**	**<0.001**	**<0.001**	**<0.001**	**<0.001**	**<0.001**
	Currently typical conformation	4.67 (2.93)	3.61 (2.44)	2.58 (1.98)	3.28 (2.31)	178.47	**<0.001**	**<0.001**	**<0.001**	**<0.001**	**<0.001**	0.133	**<0.001**
	Super extreme conformation	3.21 (2.86)	2.32 (2.25)	1.76 (1.66)	2.09 (2.12)	104.74	**<0.001**	**<0.001**	**<0.001**	**<0.001**	**<0.001**	0.244	**0.043**
How healthy does each dog look to you personally?	Less extreme conformation	7.68 (2.29)	7.26 (2.34)	5.97 (2.54)	6.46 (2.43)	110.63	**<0.001**	**0.010**	**<0.001**	**<0.001**	**<0.001**	**<0.001**	**0.016**
	Currently typical conformation	5.23 (2.72)	4.24 (2.43)	3.60 (2.25)	3.94 (2.36)	97.60	**<0.001**	**<0.001**	**<0.001**	**<0.001**	**<0.001**	0.198	0.076
	Super extreme conformation	3.54 (2.93)	2.72 (2.50)	2.40 (2.18)	2.54 (2.45)	45.98	**<0.001**	**<0.001**	**<0.001**	**<0.001**	**0.029**	0.459	0.463
How ethically do you think each of these dogs has been bred?	Less extreme conformation	7.12 (2.75)	6.99 (2.70)	5.65 (2.82)	5.87 (2.75)	68.91	**<0.001**	0.495	**<0.001**	**<0.001**	**<0.001**	**<0.001**	0.340
	Currently typical conformation	4.23 (2.52)	3.45 (2.13)	2.83 (1.96)	3.06 (1.96)	92.98	**<0.001**	**<0.001**	**<0.001**	**<0.001**	**<0.001**	0.055	0.186
	Super extreme conformation	2.50 (2.37)	1.88 (1.78)	1.70 (1.58)	1.77 (1.75)	41.07	**<0.001**	**<0.001**	**<0.001**	**<0.001**	0.084	0.515	0.617
How much would you personally like to own each of these dogs?	Less extreme conformation	5.13 (3.36)	4.32 (3.19)	2.64 (2.27)	3.67 (2.72)	197.35	**<0.001**	**<0.001**	**<0.001**	**<0.001**	**<0.001**	**0.011**	**<0.001**
	Currently typical conformation	3.50 (2.94)	2.38 (2.10)	1.59 (1.40)	2.21 (1.93)	206.82	**<0.001**	**<0.001**	**<0.001**	**<0.001**	**<0.001**	0.363	**<0.001**
	Super extreme conformation	2.55 (2.61)	1.68 (1.72)	1.28 (1.10)	1.66 (1.74)	124.50	**<0.001**	**<0.001**	**<0.001**	**<0.001**	**<0.001**	0.915	**0.004**

*Note*: Highlighted in bold are results that are statistically significant.

Abbreviation: LSD, Fisher's least significant difference.

**TABLE 5:** Comparison of the views of UK owners of purebred brachycephalic, crossbred brachycephalic and non‐brachycephalic dogs when asked a series of questions relating to images of Pugs with varying levels of extreme conformation.

		Mean score from 1 to 10 (standard deviation)						Post hoc comparison (LSD), *p*‐value					
Pug	Image category	Owners of purebred brachy‐cephalic dogs	Owners of crossbred brachy‐cephalic dogs	Owners of non‐brachy‐cephalic dogs	Non‐owners	*F*‐statistic	One‐way ANOVA, *p*‐value	Purebred versus crossbred brachy‐cephalic	Purebred versus non‐ brachy‐cephalic	Purebred versus non‐owner	Crossbred versus non‐ brachy‐cephalic	Crossbred versus non‐owner	Non‐brachy‐cephalic versus non‐owner
How happy does each dog make you feel when you look at them?	Less extreme conformation	7.15 (2.79)	7.00 (2.76)	5.62 (2.56)	6.35 (2.56)	74.43	**<0.001**	0.393	**<0.001**	**0.004**	**<0.001**	**0.002**	**<0.001**
	Currently typical conformation	5.44 (3.13)	4.23 (2.76)	2.95 (2.08)	3.58 (2.26)	219.47	**<0.001**	**<0.001**	**<0.001**	**<0.001**	**<0.001**	**0.006**	**0.001**
	Super extreme conformation	3.15 (2.87)	2.56 (2.57)	1.74 (1.61)	2.07 (1.97)	98.56	**<0.001**	**<0.001**	**<0.001**	**<0.001**	**<0.001**	**0.015**	**0.046**
How attractive does each dog look to you personally?	Less extreme conformation	6.47 (2.95)	6.45 (2.98)	4.72 (2.68)	5.54 (2.80)	99.54	**<0.001**	0.954	**<0.001**	**<0.001**	**<0.001**	**<0.001**	**<0.001**
	Currently typical conformation	5.29 (3.29)	3.79 (2.82)	2.43 (1.96)	3.02 (2.19)	290.59	**<0.001**	**<0.001**	**<0.001**	**<0.001**	**<0.001**	**0.001**	**0.002**
	Super extreme conformation	3.09 (2.80)	2.41 (2.41)	1.58 (1.46)	1.98 (2.02)	126.04	**<0.001**	**<0.001**	**<0.001**	**<0.001**	**<0.001**	**0.021**	**0.012**
How healthy does each dog look to you personally?	Less extreme conformation	7.49 (2.54)	7.42 (2.41)	6.21 (2.46)	6.52 (2.56)	67.07	**<0.001**	0.692	**<0.001**	**<0.001**	**<0.001**	**<0.001**	0.131
	Currently typical conformation	4.97 (2.77)	3.78 (2.34)	2.86 (1.94)	3.24 (2.25)	187.04	**<0.001**	**<0.001**	**<0.001**	**<0.001**	**<0.001**	**0.010**	**0.036**
	Super extreme conformation	2.72 (2.42)	2.16 (2.08)	1.68 (1.51)	1.96 (1.89)	66.440	**<0.001**	**<0.001**	**<0.001**	**<0.001**	**<0.001**	0.241	0.060
How ethically do you think each of these dogs has been bred?	Less extreme conformation	6.96 (2.92)	6.84 (2.70)	5.85 (2.69)	6.47 (2.75)	35.82	**<0.001**	0.184	**<0.001**	0.117	**<0.001**	0.066	**0.006**
	Currently typical conformation	4.63 (2.90)	3.52 (2.43)	2.68 (1.96)	3.04 (2.20)	152.26	**<0.001**	**<0.001**	**<0.001**	**<0.001**	**<0.001**	**0.031**	**0.048**
	Super extreme conformation	2.57 (2.46)	2.08 (2.03)	1.63 (1.53)	1.74 (1.71)	53.60	**<0.001**	**<0.001**	**<0.001**	**<0.001**	**<0.001**	0.057	0.473
How much would you personally like to own each of these dogs?	Less extreme conformation	5.53 (3.37)	5.16 (3.45)	3.26 (2.54)	4.40 (2.74)	120.89	**<0.001**	0.309	**<0.001**	**<0.001**	**<0.001**	**0.002**	**<0.001**
	Currently typical conformation	4.39 (3.57)	2.95 (2.64)	1.63 (1.48)	2.27 (2.06)	310.83	**<0.001**	**<0.001**	**<0.001**	**<0.001**	**<0.001**	**0.002**	**<0.001**
	Super extreme conformation	2.57 (2.77)	1.86 (2.00)	1.25 (1.02)	1.68 (1.77)	125.15	**<0.001**	**<0.001**	**<0.001**	**<0.001**	**<0.001**	0.264	**0.002**

*Note*: Highlighted in bold are results that are statistically significant.

Abbreviation: LSD, Fisher's least significant difference.

**TABLE 6:** Comparison of the views of UK owners of brachycephalic purebred, brachycephalic crossbred and non‐brachycephalic dogs when asked a series of questions relating to images of English Bulldogs with varying levels of extreme conformation.

		Mean score from 1 to 10 (standard deviation)						Post hoc comparison (LSD), *p*‐value					
English Bulldog	Image category	Owners of purebred brachy‐cephalic dogs	Owners of crossbred brachy‐cephalic dogs	Owners of non‐brachy‐cephalic dogs	Non‐owners	*F*‐statistic	One‐way ANOVA, *p*‐value	Purebred versus crossbred brachy‐cephalic	Purebred versus non‐ brachy‐cephalic	Purebred versus non‐owner	Crossbred versus non‐ brachy‐cephalic	Crossbred versus non‐owner	Non‐brachy‐cephalic versus non‐owner
How happy does each dog make you feel when you look at them?	Less extreme conformation	6.66 (2.84)	6.19 (2.74)	4.73 (2.56)	5.36 (2.62)	117.26	**<0.001**	**0.009**	**<0.001**	**<0.001**	**<0.001**	**<0.001**	**0.004**
	Currently typical conformation	5.57 (2.91)	4.38 (2.67)	3.18 (2.16)	3.74 (2.33)	203.11	**<0.001**	**<0.001**	**<0.001**	**<0.001**	**<0.001**	**0.006**	**0.005**
	Super extreme conformation	4.32 (3.30)	3.06 (2.77)	2.23 (2.01)	2.45 (2.16)	148.55	**<0.001**	**<0.001**	**<0.001**	**<0.001**	**<0.001**	**0.010**	0.273
How attractive does each dog look to you personally?	Less extreme conformation	5.99 (3.03)	5.39 (3.01)	4.03 (2.62)	4.66 (2.79)	108.81	**<0.001**	**0.001**	**<0.001**	**<0.001**	**<0.001**	**0.008**	**0.005**
	Currently typical conformation	5.19 (2.99)	3.93 (2.74)	2.71 (2.07)	3.28 (2.41)	218.83	**<0.001**	**<0.001**	**<0.001**	**<0.001**	**<0.001**	**0.006**	**0.004**
	Super‐extreme conformation	4.14 (3.26)	2.79 (2.64)	2.00 (1.88)	2.28 (2.13)	168.26	**<0.001**	**<0.001**	**<0.001**	**<0.001**	**<0.001**	**0.026**	0.144
How healthy does each dog look to you personally?	Less‐extreme conformation	7.06 (2.56)	6.50 (2.56)	5.46 (2.48)	5.88 (2.50)	85.90	**<0.001**	**0.001**	**<0.001**	**<0.001**	**<0.001**	**0.013**	**0.040**
	Currently typical conformation	5.33 (2.63)	4.38 (2.45)	3.46 (2.12)	3.78 (2.25)	135.55	**<0.001**	**<0.001**	**<0.001**	**<0.001**	**<0.001**	**0.007**	0.092
	Super extreme conformation	3.64 (2.79)	2.78 (2.43)	2.17 (1.87)	2.23 (2.01)	92.83	**<0.001**	**<0.001**	**<0.001**	**<0.001**	**<0.001**	**0.010**	0.749
How ethically do you think each of these dogs has been bred?	Less extreme conformation	6.53 (2.85)	6.31 (2.75)	5.20 (2.65)	5.78 (2.73)	55.26	**<0.001**	0.224	**<0.001**	**0.002**	**<0.001**	**0.049**	**0.009**
	Currently typical conformation	4.97 (2.66)	3.99 (2.39)	3.18 (2.11)	3.54 (2.34)	123.92	**<0.001**	**<0.001**	**<0.001**	**<0.001**	**<0.001**	**0.046**	0.056
	Super extreme conformation	3.64 (2.93)	2.75 (2.61)	2.17 (1.97)	2.18 (2.09)	84.30	**<0.001**	**<0.001**	**<0.001**	**<0.001**	**<0.001**	**0.011**	0.935
How much would you personally like to own each of these dogs?	Less extreme conformation	4.42 (3.28)	3.98 (3.10)	2.65 (2.32)	3.56 (2.63)	99.26	**<0.001**	**0.014**	**<0.001**	**<0.001**	**<0.001**	0.107	**<0.001**
	Currently typical conformation	3.79 (3.03)	2.81 (2.50)	1.81 (1.63)	2.41 (2.00)	177.66	**<0.001**	**<0.001**	**<0.001**	**<0.001**	**<0.001**	0.055	**<0.001**
	Super extreme conformation	3.12 (3.01)	2.05 (2.08)	1.44 (1.31)	1.77 (1.64)	159.96	**<0.001**	**<0.001**	**<0.001**	**<0.001**	**<0.001**	0.136	**0.036**

*Note*: Highlighted in bold are results that are statistically significant.

Abbreviation: LSD, Fisher's least significant difference.

For all questions, for all degrees of conformational extreme, the same pattern was repeated: owners of purebred brachycephalic dogs consistently scored every image highest, followed by the owners of crossbred brachycephalic dogs, then non‐owners, with owners of non‐brachycephalic dogs giving every image the lowest score (Table 4).

The responses did not differ statistically significantly between owners of crossbred brachycephalic dogs and non‐owners when scoring the typical and super extreme dogs in all question categories. Owners of non‐brachycephalic dogs and non‐owners showed no significant differences in their scores for all conformational types when asked about how ethically they had been bred and for the typical and super extreme dogs when asked about health.

Thirteen variables assessed using univariable linear regression for their association with the outcome measure ‘image score’ were liberally associated (*p* < 0.2). Following multivariable modelling, 11 variables were retained in the final model (Table 7).

**TABLE 7:** Final multivariable model of the effect of various factors on participants’ image scores.

Variable	Category	Co‐efficient	Standard error	95% confidence interval	*p*‐Value
Extremeness of conformation in image	Less extreme	Baseline			
	Current typical	**‒2.08**	0.01	‒2.11 to ‒2.05	**<0.001**
	Super extreme	**‒3.19**	0.01	‒3.22 to ‒3.16	**<0.001**
Attribute participants were asked about	Happiness	Baseline			
	Attractiveness	**‒0.46**	0.02	‒0.50 to ‒0.43	**<0.001**
	Healthiness	**0.22**	0.02	0.18–0.25	**<0.001**
	Ethically bred	**‒0.16**	0.02	‒0.20 to ‒0.12	**<0.001**
	Willingness to own	**‒1.49**	0.02	‒1.53 to ‒1.46	**<0.001**
Type of dog owned by participant	Purebred brachycephalic	Baseline			
	Crossbred brachycephalic	**‒0.77**	0.02	‒0.82 to ‒0.73	**<0.001**
	Non‐brachycephalic	**‒1.67**	0.01	‒1.70 to ‒1.64	**<0.001**
Whether the participant works in the animal sector	No	Baseline			
	Yes	**‒0.59**	0.02	‒0.61 to ‒0.55	**<0.001**
Participant household demographic	Adults only	Baseline			
	Adults with children	**0.15**	0.02	0.11–0.19	**<0.001**
	Lives alone	**‒0.13**	0.02	‒0.17 to ‒0.19	**<0.001**
Participant age (years)	18‒24	**0.41**	0.03	0.34–0.48	**<0.001**
	25‒34	**0.17**	0.02	0.12–0.21	**<0.001**
	35‒44	0.07	0.02	0.03–0.12	0.576
	45‒54	Baseline			
	55‒64	**‒0.22**	0.02	‒0.26 to ‒0.19	**<0.001**
	65+	**‒0.35**	0.02	‒0.39 to ‒0.31	**<0.001**
Breed in image	French Bulldog	Baseline			
	Pug	‒0.04	0.01	‒0.07 to ‒0.01	0.468
	English Bulldog	**0.01**	0.01	‒0.18 to 0.04	**0.011**
Number of dogs currently owned by participant	1	Baseline			
	0	‒0.05	0.03	‒0.17 to ‒0.03	0.078
	2	**0.03**	0.02	‒0.04 to 0.06	**0.050**
	3+	0.05	0.02	0.01–0.09	0.451
Whether the participant grew up with a dog	No	Baseline			
	Yes	**0.09**	0.01	0.07–0.12	**<0.001**
Participant gender	Female	Baseline			
	Male	**0.17**	0.02	0.13–0.22	**<0.001**
Participant housing type	3+ bedroom home	Baseline			
	Flat/apartment	**0.61**	0.02	0.31–0.92	**<0.001**
	1‒2 bedroom home	**0.15**	0.02	0.12–0.19	**<0.001**

*Note*: Highlighted in bold are significant results.

In the final model, after accounting for the effects of the other variables assessed, the extremeness of the conformation of the dog in the image and the type of dog owned by the participant were associated with the largest differences.

Extremeness of conformation had the greatest effect on image score, with both the super extreme and currently typical images scoring significantly lower than the less extreme images (by 3.19 points for the super extreme and 2.08 points for the currently typical). The dog type that the owner chose to acquire also had a significant effect on the image scores, with owners of non‐brachycephalic dogs and crossbred brachycephalic dogs scoring the images lower than the owners of purebred brachycephalic dogs (by 0.77 points for crossbred brachycephalic dog owners and 1.67 points for non‐brachycephalic dog owners).

### Participants' views on whether the dogs in the images are purebred

Most participants agreed that at least one of the dogs presented for each set of three images was purebred for all three breeds: around one in 13 participants selected ‘none of the above’ as purebred for the English Bulldog images (7.5%) and the Pug images (7.7%), but one in five participants selected this option for the French Bulldog images (19.5%). The image that had been digitally modified to have a less extreme conformation than is currently typical was considered purebred by around one in three participants for the English Bulldog (28.7%) and Pug (33.4%), and for two in five participants for the French Bulldog (43.5%). Most participants (70.1%) considered the super extreme dog to be purebred for the English Bulldog, compared with less than half for the Pug (45.8%) and French Bulldog (40.2%) (Table 8).

**TABLE 8:** Frequency of participants who considered digitally altered images of English Bulldogs, Pugs and French Bulldogs to represent a purebred dog.

Type	Number of participants who considered the image to represent a purebred dog (%)		
	English Bulldog (*n* = 3635)	Pug (*n* = 3635)	French Bulldog (*n* = 3733)
None of the dogs in the three images were purebred	269 (7.5)	280 (7.7)	729 (19.5)
Less extreme dog	1021 (28.7)	1214 (33.4)	1625 (43.5)
Typical dog	1396 (39.2)	2354 (64.8)	1680 (45.0)
Super extreme dog	2499 (70.1)	1664 (45.8)	1502 (40.2)

## DISCUSSION

Physical appearance is consistently reported as highly important in dog owners' choice to acquire a certain breed,^35^ with this being particularly important for owners of brachycephalic dogs.^36^, ^37^ In the current study, we used AI‐generated images to identify that the UK public exhibit a strong preference for less extreme versions of French Bulldogs, Pugs and English Bulldogs when given a choice. These are the three most common brachycephalic breeds in the UK, and they commonly suffer from severe health problems linked to their conformation.^28^, ^29^, ^30^ Across all three breeds, participants in the current study consistently showed a progressive, significant trend in rating the less extreme conformations higher for inspiring happiness, attractiveness, perceived health, ethical breeding and desire to own. Previous research has suggested that baby‐schema features, such as large, wide‐set eyes, that are common in brachycephalic dogs increase owner preference for these types of dogs.^6^ However, that study did not assess levels of preference across varying degrees of brachycephaly, all of which might meet the baby‐schema criteria to some extent.^9^

The current study indicated that less extreme variants of the French Bulldog, Pug and English Bulldog are considered more desirable by the public. However, producing less extreme variants of these typically extreme brachycephalic breeds will require a shift in perception of the sanctity of breed ‘purity’ over many generations, and commitment beyond the current breed standards, which do not appear sufficient, even with some recent amendments, to deter breeders’ selection of extreme conformation. Although documented trends in variations of the extremity of the brachycephalic phenotype are scant, examples of the less extreme conformations depicted in the current study appear uncommon in studied populations of existing purebred brachycephalic dogs.^11^, ^53^ Rapid moves towards less extreme conformation among kennel club‐registered dogs would likely require a return to broad acceptance of outcrossing as a valuable breeding tool.

Outcrossing describes breeding between dogs of different breeds, or between dogs of the same breed but with limited shared ancestry, with the goal of changing the conformation or genetic diversity of the parent breed of interest.^54^ Outcrossing has been used to augment genetic diversity in dog populations threatened by severe inbreeding and extinction, such as the Norwegian Lundehund,^55^ and to decrease the risk of breed‐specific disorders with a genetic origin, such as progressive retinal atrophies.^56^ Outcrossing has also been used to move long‐established extreme breeds towards less extreme versions by ‘breeding in’ more moderate types of dogs.^56^, ^57^, ^58^ However, it is worth noting that current knowledge on the relative health of brachycephalic outcrosses compared to their progenitor breeds is limited. One study explored the comparative health of outcrossed brachycephalic dogs by comparing purebred Pugs (*n* = 47) with ‘Retropugs’ (*n* = 7), a Pug‐type originally created in Germany by crossing purebred Pugs with Parson Russell Terriers, which exhibit a less extreme brachycephalic conformation than their purebred counterparts. In that study, Retropugs demonstrated less impairment of respiratory function compared to the purebred Pugs.^57^ Although that study was small and the health effects of outcrossing in brachycephalic dogs require further exploration, it does suggest that potential health gains are possible from strategic outcrossing, in a more rapid and marked manner than is possible via within‐breed selection. Selecting towards less extreme conformations of currently extreme breeds via outcrossing would also increase genetic diversity, which is a recognised issue in some brachycephalic breeds.^59^, ^60^ The feasibility of using outcrossing to move towards less extreme conformations depends upon public acceptance of a shift away from more ‘pure’ bloodlines and a high demand for these dogs. Owning a dog with both purebred status and high predictability of their future size, appearance and temperament is seen as desirable for potential dog owners when deciding on a type of puppy to acquire.^43^, ^44^, ^45^ However, demand may be changing, with a recent study on the perceptions of Australian participants on their ideal dog revealing that participants mostly showed no preference regarding whether their dog was purebred or not (73%), with 19.5% preferring purebred dogs and 7.4% preferring mixed breed or designer (purposefully crossbred) dogs.^61^ Furthermore, so‐called ‘designer crossbreeds’, such as Poodle‐crosses, have soared in popularity in recent years in the UK, indicating that purity is not a deterrent for many dog owners, and indeed may be considered undesirable.^3^ Whether this applies to owners of brachycephalic dogs requires further exploration.

Owners of purebred brachycephalic dogs consistently gave higher scores for inspiring happiness, attractiveness, health, ethical breeding and desire to own across all extremeness categories. Intractability among owners of extreme brachycephalic dogs (i.e., nothing could put them off) has recently been demonstrated, which was predicted by a strong preference for flat faces as a highly desirable aesthetic characteristic and incorrect beliefs that brachycephaly did not negatively impact lifespan.^33^ This intractability of acquisition behaviour may be explained by the complex relationship between relative sensitivity to the baby schema^62^ and the degree of emotional attachment between owner and pet, which can amplify cognitive dissonance when these owners are faced with information that may conflict with their views, for example, regarding the poor health status of brachycephalic breeds.^63^ However, even current owners of brachycephalic dogs in the current study showed a clear preference for less extreme conformations over the typical and super extreme types of dogs when given free choice. This suggests that even among enthusiasts of brachycephalic breeds, there is acceptance and preference for less extreme features. This suggests that a shift towards normalisation of less extreme types of French Bulldogs, Pugs and English Bulldogs would not deter even committed current owners from appreciating ownership of these breeds while also reducing welfare issues in these dogs. The current results provide valuable insight for kennel clubs and breed clubs that breed standards can be revised to remove all promotion of extreme conformation without losing the individuality of, and desire for, these breeds. The strong and consistent preference for less extreme variants of these brachycephalic dogs also shown by participants who are not currently owners of brachycephalic breeds demonstrates preference in potential future owners. This is of relevance as purchasers of brachycephalic breeds are often acquiring these breeds for the first time,^36^ and thus, the non‐brachycephalic owners studied may choose to purchase a brachycephalic breed in the future. In addition, non‐owners of brachycephalic breeds may also assert social influence over acquisition decisions^34^ and shape general public attitudes towards their ownership, including what degree of brachycephaly is considered acceptable (if any).

However, care should be taken when interpreting participants’ stated acquisition intentions, as what participants say in a survey does not necessarily reflect their ultimate actions (i.e., the ‘intention‒behaviour gap’),^64^ and human behaviour change interventions may be needed to influence future purchasing behaviour.^65^ The COM‐B behavioural change framework suggests that three interwoven concepts—capability, motivation and opportunity—interact to generate behavioural change.^65^ The preference for moderate conformations documented in this study supports the motivation domain of this framework (likely reflecting reduced automatic motivation in this population, for example, that they are less susceptible to the emotional ‘cute response’ of the paedomorphic appearance of extreme brachycephalic dogs thought to be a driver of their acquisition^9^); however, buyer reflective motivation could be enhanced by providing prospective owners with an evidence base regarding the benefits of outcrossed brachycephalic dogs to enhance their motivation (e.g., health and welfare benefits for dogs, financial and emotional implications for owners), an area currently lacking robust data. Further work is required regarding the potential for harnessing the capability and opportunity domains. For example, harnessing opportunity likely requires enhanced physical opportunity, for example, by promoting breeders to produce more outcrosses exhibiting less extreme conformation, and ensuring that they meet buyer expectations regarding acquisition motivators such as ethical practice, behaviour and health (and rewarding breeders who do as such), or by restricting opportunities to purchase dogs with the most extreme conformations via legislation such as breed bans, as seen in other countries such as the Netherlands. Furthermore, influencing social opportunity requires a shift in societal attitudes towards breeding for extreme conformation to create societal norms on what an innately healthy dog looks like, which could be influenced by messaging from key stakeholders. Finally, enhancing capability requires an informed consumer base, and could be improved by providing clear, accessible information to buyers on the welfare consequences of extreme conformation to maximise psychological capability, including myth busting of ‘normalised’ pathologies in extreme brachycephalic dogs^33^ and dogma surrounding the benefits of pure breeding, alongside clear resources to help buyers identify dogs with healthy conformations to improve physical capability, for example, hosted on key puppy selling platforms.

The UK Kennel Club claims to register 30% of all UK dogs, but that only 2% of dogs have ever entered into a dog show,^66^ highlighting that a large pet dog market exists outside the constraints of breed standards, and therefore representing a big opportunity for improving canine welfare. The production of dogs that meet the public demand for less extreme ‘pet’ brachycephalic dogs could usher in a new era of moderate versions of these longstanding French Bulldog, Pug and English Bulldog breeds with superior aesthetics and health compared to the more extreme current versions, even if show ring aesthetics remain relatively intractable to change. Regrettably, breed show judges have been reported as expressing more reluctance for actions designed to improve the health of brachycephalic dogs, such as education of breeders and puppy buyers, examinations of breeding animals, registration of BOAS surgeries and development of objective measures for assessment, compared with owners, veterinarians and breeders.^67^ Collaboration across stakeholders is important when attempting to implement change. For example, the Horse Trust's ‘Weigh to Win’ initiative tackling equine obesity is reportedly most effective when there is strong engagement from both owners and show judges.^68^ Further studies of the barriers to change are needed to develop effective interventions for this community. Furthermore, participatory engagement with the dog showing community is likely required to achieve buy‐in on future interventions aimed at moderating phenotype, with tangible benefits to breeders and judges who adhere to recommended practices. The public portrayal of dogs with extreme conformation has long been of concern to relevant bodies, both nationally and internationally, such as the British Veterinary Association, Brachycephalic Working Group and International Collaborative on Extreme Conformations in Dogs, who have long campaigned for the cessation of the use of dogs with extreme conformation in advertising and the media.^10^, ^69^, ^70^ Further change is needed to prevent the normalisation of such extreme conformations and to make the depiction of dogs in media and advertising more welfare friendly.

With the perception of owning a purebred remaining an important factor in dog acquisition for many owners,^43^, ^44^, ^45^ the current study has revealed novel and exciting insights into how changes to the conformation of certain dog breeds can alter public perceptions on purebred status. The images digitally modified to be less extreme were still accepted as representing a purebred dog by a substantial proportion of participants (43.5% French Bulldog, 33.4% Pug and 28.7% English Bulldog), suggesting public openness to embracing less extreme versions of these three breeds as still being purebred, provided the dogs retain sufficient breed‐recognisable traits. Although a minority of participants considered none of the dogs in the images as purebred (19.5% French Bulldog, 7.7% Pug and 7.5% English Bulldog), this was notably higher for French Bulldogs. This high level of rejection of all three images as being purebred could reflect the accuracy of the images created to represent this breed in comparison to real‐life examples, but it could also offer insight into breed recognition biases. Greater cognitive flexibility in the general public on what constitutes a purebred English Bulldog and Pug could be leveraged to promote healthier variants of these breeds while still meeting public criteria for being a distinct breed. Indeed, less extreme variants of these two breeds are already in existence, for example, the ‘Retropug’ and Bulldog variants such as the Victorian Bulldog. This mental flexibility on purebred status might be indicative of a wider public cultural shift towards prioritising the health and functional aspects of dog breeds over the potentially harmful pedigree dog breeding mantra, seen in some breeds with extreme conformations, that breed aesthetics always trump health issues.

Across the three breeds, the English Bulldog had the highest proportion of participants concluding that the super extreme version was purebred. During the production of the AI images for the current study, the images for the English Bulldog were the most challenging to manipulate into the three variants of extreme conformation. This could suggest that the imagery corpus of English Bulldogs available for AI technology to draw upon is more varied, that is, the background population of English Bulldogs may contain more phenotypic variation, leading to greater challenge in producing specific changes. The English Bulldog images also differ from the Pug and French Bulldog as they have a more forward‐facing stance and a more natural background, which may have contributed to differences. It has been demonstrated that UK English Bulldogs show more variation in muzzle length than some other brachycephalic breeds, such as Pugs and French Bulldogs,^11^ which suggests that an English Bulldog with an increased muzzle length could still retain enough recognisable breed traits to appeal while reducing the risk of conformational disease. However, given conformation‐related disorders for English Bulldogs are not limited to just muzzle length, outcrossing to introduce an overall healthier conformation (e.g., a healthy jaw shape avoiding the currently common prognathism in the breed, smaller relative skull size to reduce birthing problems) would likely still be necessary to protect their welfare.^23^, ^28^

The current study had some recognised limitations. Participation was limited to UK adults only; thus, the results may not generalise well internationally. As is common in surveys more generally, the current study included higher participation from higher socioeconomic background,^71^, ^72^ female^71^, ^73^ and younger^73^ demographics, with owners of brachycephalic dogs previously demonstrated to often be younger, more likely to be living with children and more likely to be living in a smaller property.^36^ To maximise dissemination to a large and varied audience, the survey was distributed across several different social media platforms and groups and circulated by a large array of different organisations. However, the proportion of participants who worked in the animal sector may have skewed findings towards more positive perceptions of less extreme conformations. Studies on brachycephalic cats that included a similar proportion of animal/veterinary care professionals (18.7%) demonstrated that owners from this group rated brachycephalic cats significantly lower than participants who did not work in these professions.^46^

Current and previous dog owners were grouped together in the analyses to improve category sample size and therefore study power. This grouping method may have affected the accuracy with which participants' current opinions could be derived from the results, as those who previously owned a brachycephalic dog but chose not to acquire another brachycephalic dog were grouped with current brachycephalic owners in the analysis. However, this is likely an unusual occurrence due to the documented high breed loyalty among owners of brachycephalic dogs.^33^, ^34^, ^38^ Although participants were provided with definitions of ‘brachycephalic’, ‘purebred’ and ‘crossbred’, no distinction was made between pedigree and non‐pedigree subsets of purebred dogs, which may have led to some nuance being missed in the responses. Social desirability bias is a documented limitation in surveys, but this was mitigated where possible through reassurances of anonymity and confidentiality and through the use of neutral questioning techniques.^74^ The coat colour of the dogs in the images was largely based upon those accepted in the UK Kennel Club breed standards^40^; however, the coat colour of the AI‐generated French Bulldog images was lighter than the fawn coat recognised as an acceptable colour in the Kennel Club breed standard. Coat colour may have influenced participants’ perceptions of the images, although evidence on colour preference in acquisition/adoption is mixed.^75^, ^76^ This effect was mitigated by using the same coat colour for all three images, to avoid a relative effect between the three conformational types.

## CONCLUSIONS

These results report a consistent and strong preference within the UK general public for less extreme conformation variants across three extreme brachycephalic breeds: the French Bulldog, Pug and English Bulldog. These findings offer major welfare opportunities to reverse the current normalisation of extreme body shapes that are linked to serious health concerns for these breeds. It appears that current breed standards could be amended to remove explicit or implicit promotion of extreme conformations without reducing the popularity of the less extreme variants of kennel club‐registered dogs while promoting better health. Furthermore, production of less extreme variants for the pet market is likely to achieve high demand while also promoting less welfare harm than with the current more extreme variants. UK puppy buyers might actively acquire dogs with less extreme body shapes in preference to more extreme types, if only these less extreme options were made available. Outcrossing is likely required to achieve the less extreme variants considered desirable in this study, which could achieve major positive impacts for breed health and welfare. The UK public, including current owners of brachycephalic dogs, appears to have finally reached a tipping point, and may now favour less extreme variants of previously extreme breeds.

## AUTHOR CONTRIBUTIONS

*Conceptualisation*: Rowena M.A. Packer. *Methodology*: all authors. *Questionnaire development*: Rowena M.A. Packer, Elizabeth Youens, Dan G. O'Neill, Zoe Belshaw, Johanna Neufuss, and Mickey S. Tivers. *Images*: Sayaka Mochizuki. *Data analysis*: Elizabeth Youens, Rowena M.A. Packer, and Dan G. O'Neill. *Writing—original draft preparation*: Elizabeth Youens, Rowena M.A. Packer, and Dan G. O'Neill. *Writing—review and editing*: all authors. All authors have read and agreed to the published version of the manuscript.

## CONFLICT OF INTEREST STATEMENT

The authors declare they have no conflicts of interest.

## ETHICS STATEMENT

Ethical approval was granted from the Social Science Research Ethical Review Board at Royal Veterinary College (URN SR2023‐0162).

## Supporting information



Supporting Information

Supporting Information

## Data Availability

The data that support the findings of this study are available from the corresponding author upon reasonable request.
