# Supplementary figures and images for: Beauty versus the beast: The UK public prefers less‐extreme body shapes in brachycephalic dog breeds
Source: Vet Rec. 2025 Jul 4;197(6):e5671. doi: 10.1002/vetr.5671 (PMC12447671; doi:10.1002/vetr.5671)

Appendix 1: Advertising poster posted on social media


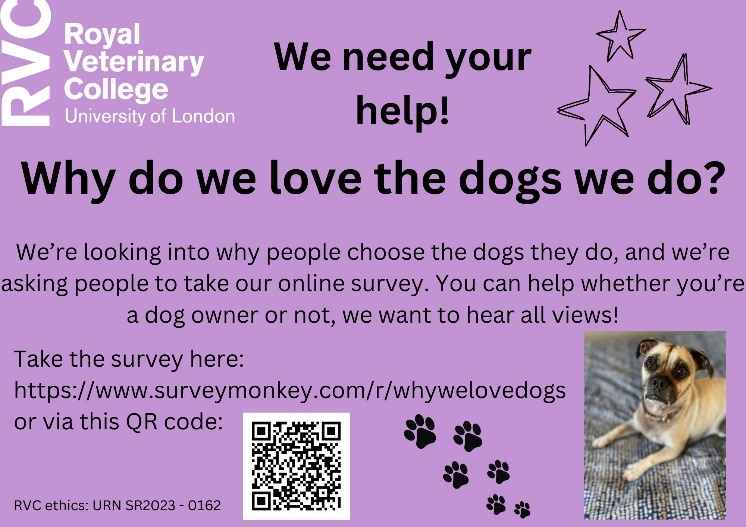

Supplement: Supplementary file 1 — Supporting Information [file VETR-197-e5671-s001.docx]
