# Supplementary material for: Beauty versus the beast: The UK public prefers less‐extreme body shapes in brachycephalic dog breeds
Source: Vet Rec. 2025 Jul 4;197(6):e5671. doi: 10.1002/vetr.5671 (PMC12447671; doi:10.1002/vetr.5671)
Supplement: Supplementary file 2 — Supporting Information [file VETR-197-e5671-s002.docx]

Appendix 2: Results: Owner demographics

**Age group frequency and percentage of survey participants**

| **Age group (years)** | **Frequency** | **Percentage** |
| --- | --- | --- |
| 18-24 | 250 | 5.1 |
| 25-34 | 895 | 18.3 |
| 35-44 | 837 | 17.1 |
| 45-54 | 1095 | 22.4 |
| 55-64 | 1030 | 21.0 |
| 65-74 | 645 | 13.2 |
| 75 and over | 147 | 3.0 |
| **Total** | **4899** | **100.0** |

**Frequency and percentage of accommodation types for survey participants**

| **Type of home** | **Frequency** | **Percentage** |
| --- | --- | --- |
| Flat/apartment/maisonette | 467 | 9.5 |
| 1-2 bed house or bungalow | 962 | 19.6 |
| 3+ house or bungalow | 3415 | 69.7 |
| Static home or caravan | 4 | 0.1 |
| Houseboat | 2 | 0.0 |
| Prefer not to say | 49 | 1.0 |
| **Total** | **4899** | **100.0** |

**Frequency and percentage of different household demographics of survey participants**

| **Household type** | **Frequency** | **Percentage** |
| --- | --- | --- |
| Live on my own | 815 | 16.6 |
| Live in a home with one or more adults but no children | 2865 | 58.5 |
| Live in a home with one or more children when I am the only adult | 131 | 2.7 |
| Live in a home with one or more adults and one or more children | 1028 | 21.0 |
| Live in a home with children visiting regularly | 20 | 0.4 |
| Prefer not to say | 40 | 0.8 |
| **Total** | **4899** | **100.0** |
